# Changes in Body Composition and Sexual Satisfaction Following Metabolic and Bariatric Surgery in Women with Obesity: A Prospective Study

**DOI:** 10.3390/healthcare14182954

**Published:** 2026-09-10

**Authors:** Marija Arapović, Marina Ćurlin, Marin Senčar, Željka Kanižaj Rogina, Alen Pajtak

**Affiliations:** 1 Faculty of Health Studies, University of Mostar, Zrinskog Frankopana 34, 88000 Mostar, Bosnia and Herzegovina; marina.curlin@fzs.sum.ba; 2 Department of Physiotherapy, University North, Jurja Križanića 31b, 42000 Varaždin, Croatia; apajtak@unin.hr; 3 Department of Abdominal Surgery, General Hospital Varaždin, Ivana Meštrovića Street 1, 42000 Varaždin, Croatia; marin_sencar@hotmail.com; 4 Department of Nursing, University North, Jurja Križanića 31b, 42000 Varaždin, Croatia; zkanizaj@unin.hr

**Keywords:** obesity, metabolic and bariatric surgery, body composition, sexual satisfaction, health-related quality of life, weight loss

## Abstract

Background: Obesity is associated with impaired health-related quality of life, including reduced sexual satisfaction. Metabolic and bariatric surgery leads to substantial weight loss and favorable changes in body composition; however, its association with sexual satisfaction as a health-related outcome remains insufficiently explored. The aim of this study was to examine changes in body composition and sexual satisfaction six months following metabolic and bariatric surgery. Methods: This prospective study included 40 women with obesity who underwent metabolic and bariatric surgery performed according to standard clinical indications and multidisciplinary team recommendations. Body composition parameters were assessed using bioelectrical impedance analysis, and sexual satisfaction was evaluated using the New Sexual Satisfaction Scale at baseline and six months postoperatively. Changes in anthropometric, body composition, and sexual satisfaction measures were analyzed using nonparametric statistical tests. Results: Six months after surgery, significant changes were observed in body composition and sexual satisfaction. Median body weight decreased from 119.55 to 100.80 kg, BMI from 42.15 to 35.65 kg/m^2^, and visceral fat level from 14.50 to 11.00 (all *p* < 0.001). Although absolute skeletal muscle mass decreased significantly (28.40 to 26.30 kg, *p* < 0.001), its relative proportion increased (25.35% to 25.90%, *p* = 0.002). Median total NSSS score increased from 62.50 to 69.50, with significant improvements in both NSSS subscales (all *p* < 0.001). Conclusions: Metabolic and bariatric surgery was associated with significant improvements in body composition and sexual satisfaction six months postoperatively. These findings suggest that improvements in physical health following metabolic and bariatric surgery may be accompanied by enhancements in sexual well-being.

## 1. Introduction

Obesity is recognized as one of the leading public health challenges worldwide and has reached unprecedented global proportions [1]. The prevalence of severe obesity has increased substantially over recent decades and is expected to continue rising. This growing burden reflects a complex interplay of biological, environmental, and sociocultural factors, including changes in dietary patterns, reduced physical activity, urbanization, chronic psychological stress, and disturbances of circadian rhythms [2,3]. Obesity is associated with numerous metabolic, endocrine, reproductive, and psychosocial consequences. It also substantially impairs health-related quality of life and places a considerable burden on healthcare systems [4,5,6,7,8].

Metabolic and bariatric surgery is widely accepted as one of the most effective therapeutic strategies for severe obesity, resulting in sustained and clinically meaningful weight loss [9,10]. Beyond weight reduction, these procedures induce metabolic and hormonal adaptations, including improvements in insulin sensitivity and alterations in gastrointestinal hormone secretion, bile acid metabolism, and gut microbiota composition. These changes contribute to the remission of type 2 diabetes and a reduction in cardiometabolic risk, highlighting the role of metabolic and bariatric surgery as a comprehensive metabolic intervention [11,12,13]. However, treatment success should not be evaluated solely on the basis of weight loss. Detailed assessment of body composition, including fat mass, fat-free mass, visceral adipose tissue, and skeletal muscle mass, provides a more comprehensive understanding of postoperative outcomes [14,15,16,17]. Visceral adipose tissue is metabolically active and plays an important role in insulin resistance, chronic systemic inflammation, and endocrine dysfunction [18,19,20,21]. Its reduction following metabolic and bariatric surgery has been associated with favorable metabolic and hormonal effects [22,23,24]. At the same time, substantial fat mass loss after surgery is frequently accompanied by a reduction in fat-free mass, particularly skeletal muscle mass, especially during the first postoperative year. Approximately 20–30% of total weight loss may be attributable to lean tissue loss [25,26,27]. Loss of skeletal muscle may adversely affect basal metabolic rate, functional capacity, and long-term weight maintenance. It may also increase the risk of sarcopenia, particularly in individuals with lower baseline muscle mass [28,29,30,31,32]. These findings underscore the clinical relevance of monitoring body composition changes during the postoperative period.

Beyond its somatic and metabolic consequences, obesity also affects psychosocial dimensions of health, including sexual health, which is recognized as an integral component of overall health and well-being [33,34]. Sexual satisfaction represents a key dimension of health-related quality of life, encompassing physical, emotional, and relational aspects of sexual experience. Obesity has been associated with impaired sexual functioning and reduced sexual satisfaction. Proposed mechanisms include hormonal alterations, negative body image, reduced self-esteem, and interpersonal factors [35,36,37]. Despite its clinical relevance, sexual satisfaction remains relatively under-addressed in clinical practice and research, particularly among individuals with obesity [38,39].

Although metabolic and bariatric surgery has been shown to improve obesity-associated medical problems and overall quality of life, studies simultaneously assessing changes in body composition and sexual satisfaction during the early postoperative period remain limited. Given the known sex-specific differences in body composition, hormonal regulation, and quality-of-life outcomes following metabolic and bariatric surgery, the present study focused exclusively on women. Therefore, the aim of this study was to examine changes in body composition and sexual satisfaction six months following metabolic and bariatric surgery. We hypothesized that metabolic and bariatric surgery would be associated with significant improvements in body composition parameters and sexual satisfaction at six months postoperatively.

## 2. Materials and Methods

### 2.1. Study Design and Participants

This prospective pre-post study included women with obesity who underwent metabolic and bariatric surgery between September 2024 and November 2025 at General Hospital Varaždin. Assessments were conducted at two time points: preoperatively (baseline) and six months postoperatively at University North. Participants underwent either laparoscopic sleeve gastrectomy or gastric bypass according to standard clinical indications and multidisciplinary team recommendations. Eligible participants were adult women with obesity scheduled to undergo metabolic and bariatric surgery who reported having a stable intimate partner. Participants were required to complete baseline and six-month postoperative assessments. Women without a partner at baseline, those who did not complete both assessments, and those with conditions that could compromise the reliable completion of self-reported questionnaires or independently affect sexual function were excluded. Participants with genitourinary conditions compromising sexual function were also excluded.

No a priori sample size calculation was performed. A consecutive recruitment approach was used, whereby all eligible women undergoing metabolic and bariatric surgery at the study center between September 2024 and November 2025 were invited to participate. The sample size was therefore determined by the number of eligible patients who agreed to participate during the predefined study period rather than by a predefined statistical power calculation.

A total of 44 women were initially enrolled in the study. During the six-month follow-up period, three participants were lost to follow-up because they did not attend the postoperative assessment, and one participant was excluded because she no longer had a stable intimate partner and therefore no longer met the eligibility criteria at the six-month assessment. Consequently, 40 participants completed both assessments and were included in the final analysis.

This study was conducted in accordance with the Declaration of Helsinki and was approved by the Ethics Committee of University North (Approval number: 2137-0336-07-24-1, date: 31 July 2024) and the Ethics Committee of General Hospital Varaždin (Approval number: 2186-192-38-24-7, date: 9 September 2024). Written informed consent was obtained from all participants prior to enrollment.

### 2.2. Measurement Instruments

#### 2.2.1. Sociodemographic Characteristics

Participants provided sociodemographic information including age, place of residence, educational level, and relationship status (married or in a stable non-marital partnership).

#### 2.2.2. Body Composition Assessment

Measurements were conducted at University North, Varaždin, Croatia. Body composition was assessed using the professional segmental body composition analyzer TANITA MC-780MA (Tanita Corporation, Tokyo, Japan), a medical-grade device based on multifrequency bioelectrical impedance analysis (BIA). The device uses eight electrodes and multiple frequencies to estimate body composition from measured bioelectrical impedance parameters. The TANITA MC-780MA has been evaluated against dual-energy X-ray absorptiometry (DXA) in previous research, demonstrating reasonable diagnostic performance for the assessment of low muscle mass, although absolute lean-mass estimates should be interpreted with appropriate caution [40]. The following parameters were obtained: body weight, body mass index (BMI), total body fat percentage, fat mass, visceral fat level, fat-free mass, muscle mass, skeletal muscle mass, bone mass, and sarcopenia index. Measurements were performed according to standardized manufacturer instructions to ensure consistency and reproducibility. Participants were assessed in the morning in a fasting state and were instructed to avoid intense physical activity and excessive fluid intake before assessment, in accordance with manufacturer recommendations.

#### 2.2.3. Metabolic and Bariatric Surgery

All metabolic and bariatric surgeries were performed in General Hospital Varaždin by experienced bariatric surgeons. All patients underwent either laparoscopic sleeve gastrectomy or Roux-en-Y gastric bypass according to standard clinical indications and multidisciplinary team recommendations. Of the 40 participants included in the final analysis, 34 (85.0%) underwent sleeve gastrectomy, whereas 6 (15.0%) underwent Roux-en-Y gastric bypass.

#### 2.2.4. Sexual Satisfaction Assessment

Sexual satisfaction was assessed using the New Sexual Satisfaction Scale (NSSS), developed and validated by Štulhofer and Buško [41]. The NSSS is a well-established instrument with demonstrated good psychometric properties. It evaluates subjective sexual experience across multiple dimensions, including overall sexual satisfaction, emotional intimacy, partner-related interaction, and perceived sexual functioning. Participants rated each of 20 statements on a 5-point Likert-type scale (1–5), with higher scores indicating greater sexual satisfaction. Total NSSS scores were calculated as the sum of statement responses (range 20–100), and subscale scores were computed according to the original instrument structure. In addition to the total and subscale scores, exploratory item-level analyses were performed for the 20 individual NSSS items to examine domain-specific changes following surgery.

### 2.3. Data Analysis

Statistical analyses were performed using IBM SPSS Statistics software (version 29.0; IBM Corp., Armonk, NY, USA). Descriptive statistics were used to summarize participant characteristics and study variables. Normality of data distribution was assessed using the Shapiro–Wilk test. Due to deviations from normal distribution, the nonparametric Wilcoxon signed-rank test was applied to compare paired preoperative and six-month postoperative measurements. The main body-composition and NSSS outcomes are presented as medians and interquartile ranges (IQR; 25th–75th percentile). Effect sizes (*r*) were calculated from the Z-values of the Wilcoxon signed-rank tests and are reported with 95% confidence intervals. The NSSS total score and the two predefined subscales were treated as the principal NSSS outcomes, whereas analyses of the 20 individual NSSS items were considered exploratory. For the item-level NSSS analyses, *p*-values were adjusted for multiple testing using the Benjamini–Hochberg false discovery rate (FDR) procedure. Place of residence (urban vs. rural) was included as an exploratory contextual variable because previous studies have considered residential environment as a potentially relevant sociodemographic factor in female sexual health, although findings regarding rural–urban differences have been inconsistent [42,43]. Subgroup comparisons according to place of residence were performed using the Mann–Whitney U test. Total NSSS scores and the two NSSS subscales were compared between the two groups at baseline and six months after surgery. Statistical significance was set at *p* < 0.05, and all tests were two-tailed.

## 3. Results

Of the 44 women initially enrolled, 40 completed the six-month follow-up and were included in the final analysis. Three participants were lost to follow-up because they did not attend the postoperative assessment, and one participant was excluded because she no longer met the eligibility criterion of having a stable intimate partner.

The mean age of the participants was 43.75 ± 6.53 years, with 55.0% of women aged between 40 and 49 years. A total of 57.5% of participants resided in urban areas and 42.5% in rural areas. Among participants, 82.5% of them were married, while 17.5% reported a stable non-marital partnership. With regard to educational attainment, 40.0% of participants held a master’s degree, 25.0% had completed a bachelor’s degree, and 35.0% had completed secondary education. The demographic characteristic of the participants are presented in Table 1.

At six months postoperatively, significant reductions were observed in body weight, BMI, total body fat percentage, fat mass, visceral fat level, fat-free mass, muscle mass, skeletal muscle mass, and bone mass (all *p* < 0.001). The largest effect sizes were observed for body weight, fat mass, and visceral fat level (*r* = 0.61), followed by BMI and total body fat percentage (*r* = 0.60). Absolute skeletal muscle mass decreased significantly (*r* = 0.50, *p* < 0.001), whereas the relative proportion of skeletal muscle mass increased significantly (*r* = 0.35, *p* = 0.002). No statistically significant change was observed in the sarcopenia index (*r* = 0.15, *p* = 0.194) (Table 2).

Six months after metabolic and bariatric surgery, statistically significant improvements were observed in both NSSS subscales and in the total NSSS score. The Personal Experiences Subscale increased from a median of 31.50 (IQR 29.00–35.00) preoperatively to 35.50 (IQR 31.00–43.00) at six months postoperatively (*r* = 0.60, 95% CI 0.44–0.72; *p* < 0.001). Similarly, the Partner- and Sexual Activity-Centered Subscale increased from 32.00 (IQR 30.00–37.00) to 36.50 (IQR 32.00–44.75) (*r* = 0.60, 95% CI 0.44–0.72; *p* < 0.001). The total NSSS score increased from 62.50 (IQR 60.00–72.75) to 69.50 (IQR 64.00–88.00), with an effect size of *r* = 0.62 (95% CI 0.46–0.74; *p* < 0.001) (Table 3).

In addition to the analysis of the NSSS total and subscale scores, an exploratory item-level analysis was performed to examine changes in the 20 individual NSSS items. To account for multiple testing, *p*-values were adjusted using the Benjamini–Hochberg false discovery rate (FDR) procedure. Statistically significant improvements were observed across all individual NSSS items, and all 20 items remained statistically significant after FDR correction (adjusted *p* < 0.05 for all items) (Table 4).

Subgroup comparisons according to place of residence (urban vs. rural) are presented in Table 5. Participants residing in rural areas had significantly higher total NSSS scores and higher scores on both NSSS subscales than participants from urban areas at baseline and six months after surgery (all *p* < 0.001). Descriptive statistics, Mann–Whitney U statistics, and *p*-values are presented in Table 5.

## 4. Discussion

The present study demonstrated significant changes in both body composition and sexual satisfaction six months following metabolic and bariatric surgery in women with obesity. Substantial reductions were observed in body weight, BMI, total body fat, fat mass, and visceral fat. Although absolute fat-free mass and skeletal muscle mass decreased, skeletal muscle mass increased relative to body weight. In parallel, sexual satisfaction improved significantly following surgery, with higher postoperative scores across the assessed NSSS dimensions. Overall, these findings support the study hypothesis that metabolic and bariatric surgery would be associated with significant improvements in body composition parameters and sexual satisfaction at six months postoperatively.

Of particular clinical relevance is the significant reduction in visceral fat, a key preoperative risk factor for cardiometabolic and endocrine disorders in individuals with obesity. Visceral adipose tissue exhibits high metabolic activity, and its reduction has been associated with improvements in insulin sensitivity, inflammatory processes, and overall cardiometabolic risk [18,22]. Metabolic and bariatric surgery is also associated with complex postoperative endocrine adaptations involving multiple hormonal axes [44]. These findings highlight that the physiological effects of metabolic and bariatric surgery extend beyond overall weight reduction and support the importance of considering metabolic and endocrine changes during postoperative follow-up.

In addition to the pronounced reduction in fat mass, the study demonstrated statistically significant decreases in fat-free mass and skeletal muscle mass. This pattern is consistent with previous reports showing that substantial weight loss following metabolic and bariatric surgery is frequently accompanied by loss of lean tissue, particularly during the early postoperative period [25,26,27]. Loss of skeletal muscle mass represents a clinically important challenge in post-bariatric management, as it may adversely affect basal metabolic rate, functional capacity, and long-term weight maintenance and may increase the risk of sarcopenia [28,29,30,31,32]. Previous research has also demonstrated substantial postoperative reductions in lean mass, further emphasizing the importance of monitoring body composition during rapid weight loss [45].

Beyond changes in body composition, the present findings indicate that metabolic and bariatric surgery was associated with significant improvements in subjective sexual satisfaction. Significant improvements were observed in the total NSSS score and both predefined NSSS subscales. In the exploratory item-level analysis, improvements were observed across all individual NSSS items, and all 20 item-level comparisons remained statistically significant after correction for multiple testing using the Benjamini–Hochberg FDR procedure. These improvements encompassed aspects related to personal sexual experiences, emotional openness, orgasm quality, perceived pleasure, and partner-focused aspects of sexual activity. In considering the relational context of these findings, previous qualitative research in individuals participating in bariatric surgery programmes has highlighted partners, family members, and friends as important sources of social support during the bariatric surgery process [46]. The present findings are broadly consistent with previous evidence demonstrating improvements in female sexual health following metabolic and bariatric surgery [34,47,48,49,50,51,52,53,54,55]. Much of the previous literature has focused primarily on sexual function and dysfunction, frequently using instruments such as the Female Sexual Function Index (FSFI) [47,49,51,52,53,54,55]. In contrast, the NSSS assesses subjective sexual satisfaction across both personal sexual experiences and partner-focused aspects of sexual activity, thereby extending the assessment beyond sexual function alone [40]. Although the NSSS has recently been applied in the bariatric population [50], the present prospective study evaluates within-participant changes from baseline to six months after surgery while simultaneously assessing detailed changes in body composition. This combined approach provides a broader perspective on early postoperative changes in physical and sexual well-being in women with obesity.

The present study demonstrated concurrent postoperative improvements in body composition and sexual satisfaction; however, the relationship between the magnitude of changes in body composition and changes in sexual satisfaction was not specifically examined. Therefore, the observed improvement in sexual satisfaction cannot be attributed directly to reductions in body weight, BMI, or visceral adiposity based on the present data. Previous studies have suggested that weight loss and changes following metabolic and bariatric surgery may be accompanied by improvements in body image, self-perception, and sexual self-confidence [33,36,37]; however, these potential mechanisms remain outside the scope of the present analysis. Women with obesity frequently report body-related self-consciousness and reduced sexual confidence, which may negatively influence sexual engagement [33,36,37]. The previous literature suggests that improvements in body image and physical functioning following bariatric surgery may contribute to a more positive sexual self-concept [36,37,48]. In the present study, improvements were observed not only in personal sexual experiences but also in partner-focused NSSS items, including perceived partner engagement, care, willingness, and relational balance. These findings indicate changes in the interpersonal dimension of reported sexual satisfaction, although the mechanisms underlying these changes were not assessed. The substantial increases observed across both main NSSS subscales indicate that postoperative improvements extended to overall sexual satisfaction rather than isolated aspects of sexual functioning.

The exploratory subgroup analysis according to place of residence was included to examine whether residential context might be associated with sexual satisfaction in this population. Previous studies have considered place of residence as a potentially relevant sociodemographic and contextual factor in female sexual health; however, findings regarding rural–urban differences have been inconsistent [42,43]. In the present study, participants from rural areas demonstrated significantly higher total NSSS and subscale scores than participants from urban areas both at baseline and six months postoperatively. Importantly, these differences were already present before surgery. Therefore, the findings should not be interpreted as evidence that place of residence modifies the effect of metabolic and bariatric surgery on sexual satisfaction. Rather, they indicate pre-existing differences between the two subgroups that persisted during the early postoperative period. Given the relatively small subgroup sizes and the exploratory nature of this analysis, these findings should be interpreted cautiously and regarded as hypothesis-generating rather than confirmatory.

From a clinical perspective, the present findings highlight the potential value of a multidimensional approach to postoperative follow-up after metabolic and bariatric surgery. Assessment of treatment outcomes may extend beyond changes in body weight and BMI to include body composition, particularly the preservation of fat-free mass and skeletal muscle mass during rapid postoperative weight loss. Monitoring these parameters may help identify patients who could benefit from individualized nutritional support and strategies aimed at preserving muscle mass. In addition, the observed improvement in sexual satisfaction suggests that sexual health may represent a relevant patient-reported outcome following metabolic and bariatric surgery. When appropriate, incorporating sexual health assessment into postoperative follow-up may facilitate the identification of concerns that are not routinely captured by standard anthropometric and metabolic evaluations. Taken together, these findings support a more comprehensive, patient-centered approach in which physical and psychosocial outcomes are considered alongside conventional measures of surgical success.

### Study Limitations

Several limitations of this study should be acknowledged. The relatively small sample size, the absence of a non-surgical control group, and the six-month follow-up period limit the generalizability of the findings and constrain causal interpretation. Furthermore, the unequal distribution of surgical procedures, with only six participants (15.0%) undergoing Roux-en-Y gastric bypass compared with 34 (85.0%) undergoing sleeve gastrectomy, limited the statistical power and reliability of a separate comparison of outcomes according to surgery type. Therefore, the potential influence of surgical procedure on the observed postoperative changes could not be reliably evaluated in the present sample. Although participants provided information on existing medical conditions, these data were not included as covariates in the statistical analyses. Potentially relevant confounding factors for sexual satisfaction were not comprehensively assessed or controlled for in the present analysis. Specifically, menopausal status, hormonal therapy, medications potentially affecting sexual function, and detailed relationship-related factors were not systematically assessed. Therefore, the potential influence of these factors on the observed changes in sexual satisfaction cannot be excluded and should be considered when interpreting the findings. Sexual satisfaction was assessed using a self-reported questionnaire and may therefore be subject to reporting and social desirability bias. Despite these limitations, the prospective study design and the use of validated assessment instruments provide valuable insight into early postoperative changes in body composition and sexual well-being. Further studies with larger samples, longer follow-up periods, and comprehensive assessment of potential confounding factors are needed to clarify the long-term clinical relevance of these findings.

## 5. Conclusions

Six months following metabolic and bariatric surgery, women with obesity demonstrated significant reductions in body weight, BMI, total body fat, fat mass, and visceral fat, together with reductions in absolute fat-free mass and skeletal muscle mass. Despite the absolute loss of skeletal muscle mass, its proportion relative to body weight increased. Sexual satisfaction also improved significantly, with higher total NSSS scores and improvements across both NSSS subscales. Overall, metabolic and bariatric surgery was associated with significant changes in body composition and improved sexual satisfaction at six months postoperatively.

## Figures and Tables

**Table 1:** Demographic characteristics of the participants (*n* = 40).

Variable	Value
Age (years), mean ± SD	43.75 ± 6.53
18–29	1 (2.5%)
30–39	10 (25.0%)
40–49	22 (55.0%)
50–59	6 (15.0%)
60–69	1 (2.5%)
Place of residence, *n* (%)	
Urban	23 (57.5%)
Rural	17 (42.5%)
Relationship status, *n* (%)	
Married	33 (82.5%)
Non-marital partnership	7 (17.5%)
Level of education, *n* (%)	
Secondary education	14 (35.0%)
Bachelor’s degree	10 (25.0%)
Master’s degree	16 (40.0%)

**Table 2:** Changes in body composition parameters from baseline to six months after metabolic and bariatric surgery (*n* = 40).

Parameter	Preoperative, Median (IQR)	6 Months Postoperative, Median (IQR)	Effect Size, *r* (95% CI)	*p*-Value
Body weight (kg)	119.55 (109.38–137.78)	100.80 (90.45–115.73)	0.61 (0.45–0.73)	<0.001
Body mass index (kg/m^2^)	42.15 (39.68–46.30)	35.65 (33.60–40.18)	0.60 (0.44–0.72)	<0.001
Body fat (fatp %)	45.35 (40.90–47.95)	37.15 (31.60–42.80)	0.60 (0.44–0.72)	<0.001
Fat mass (fatm. kg)	52.80 (46.73–61.58)	37.30 (30.98–49.28)	0.61 (0.45–0.73)	<0.001
Visceral fat level (vfatl)	14.50 (12.25–16.75)	11.00 (8.25–13.00)	0.61 (0.45–0.73)	<0.001
Fat-free mass (ffm. kg)	66.60 (60.08–72.60)	62.75 (58.68–69.05)	0.43 (0.23–0.59)	<0.001
Muscle mass (pmm. kg)	63.20 (56.90–68.93)	59.55 (55.68–65.43)	0.43 (0.23–0.59)	<0.001
Skeletal muscle mass (smmm. kg)	28.40 (26.75–34.68)	26.30 (24.43–29.13)	0.50 (0.31–0.65)	<0.001
Skeletal muscle mass (%)	25.35 (22.53–28.98)	25.90 (22.50–32.13)	0.35 (0.14–0.53)	0.002
Bone mass (bonem. kg)	3.40 (3.03–3.68)	3.20 (3.00–3.48)	0.43 (0.23–0.59)	<0.001
Sarcopenia index (sarci)	9.11 (8.44–9.86)	8.95 (8.50–9.64)	0.15 (−0.07–0.36)	0.194

Data are presented as median and interquartile range (IQR; 25th–75th percentile). Effect size (*r*) was calculated from the Z-value of the Wilcoxon signed-rank test. CI, confidence interval.

**Table 3:** Changes in NSSS total and subscale scores from baseline to six months after metabolic and bariatric surgery (*n* = 40).

NSSS Domain	Preoperative, Median (IQR)	6 Months Postoperative, Median (IQR)	Effect Size, *r* (95% CI)	*p*-Value
Personal experiences subscale	31.50 (29.00–35.00)	35.50 (31.00–43.00)	0.60 (0.44–0.72)	<0.001
Partner- and sexual activity-centered subscale	32.00 (30.00–37.00)	36.50 (32.00–44.75)	0.60 (0.44–0.72)	<0.001
Total NSSS score	62.50 (60.00–72.75)	69.50 (64.00–88.00)	0.62 (0.46–0.74)	<0.001

Note: Data are presented as median and interquartile range (IQR; 25th–75th percentile). Effect size (*r*) was calculated from the Z-value of the Wilcoxon signed-rank test. CI, confidence interval; NSSS, New Sexual Satisfaction Scale.

**Table 4:** Exploratory item-level analysis of changes in individual NSSS items from baseline to six months after metabolic and bariatric surgery (*n* = 40).

Parameter NSSS	Preoperative, Median (IQR)	6 Months Postoperative, Median (IQR)	Effect Size, *r* (95% CI)	Adjusted *p*-Value
Personal sexual experiences				
Sexual arousal intensity	3.00 (3.00–3.00)	4.00 (3.00–4.00)	0.51 (0.33–0.66)	<0.001
Orgasm quality	3.00 (3.00–4.00)	4.00 (3.00–4.75)	0.54 (0.36–0.68)	<0.001
Letting go to pleasure	3.00 (3.00–4.00)	4.00 (3.00–4.00)	0.49 (0.30–0.64)	<0.001
Ability to focus	3.00 (3.00–4.00)	4.00 (3.00–4.75)	0.49 (0.30–0.64)	<0.001
Responsiveness to partner	3.00 (3.00–4.00)	4.00 (3.00–4.00)	0.47 (0.28–0.63)	<0.001
Body functioning	3.00 (3.00–4.00)	4.00 (3.00–4.00)	0.49 (0.30–0.64)	<0.001
Emotional openness	3.00 (3.00–3.75)	4.00 (3.00–4.00)	0.54 (0.36–0.68)	<0.001
Mood after sex	3.00 (3.00–4.00)	4.00 (3.00–5.00)	0.47 (0.28–0.63)	<0.001
Orgasm frequency	3.00 (2.25–4.00)	4.00 (3.00–5.00)	0.43 (0.23–0.59)	<0.001
Pleasure provided to partner	3.00 (3.00–4.00)	4.00 (3.00–4.75)	0.51 (0.33–0.66)	<0.001
Partner-focused and sexual activity				
Balance between giving and receiving	3.00 (3.00–4.00)	4.00 (3.00–4.75)	0.47 (0.28–0.63)	<0.001
Emotional openness	3.00 (3.00–4.00)	4.00 (3.00–5.00)	0.50 (0.31–0.65)	<0.001
Partner’s initiation	3.00 (3.00–4.00)	4.00 (3.00–5.00)	0.46 (0.27–0.62)	<0.001
Partner’s orgasm	3.00 (3.00–4.00)	4.00 (3.00–5.00)	0.45 (0.25–0.61)	<0.001
Partner’s engagement	3.00 (3.00–4.00)	4.00 (3.00–5.00)	0.46 (0.27–0.62)	<0.001
Partner’s care	3.00 (3.00–4.00)	4.00 (3.00–4.00)	0.47 (0.28–0.63)	<0.001
Partner’s creativity	3.00 (3.00–4.00)	4.00 (3.00–4.75)	0.47 (0.28–0.63)	<0.001
Partner’s willingness	3.00 (3.00–4.00)	4.00 (3.00–5.00)	0.51 (0.33–0.66)	<0.001
Variety of sexual activities	3.00 (3.00–4.00)	4.00 (3.00–5.00)	0.49 (0.30–0.64)	<0.001
Frequency of sexual activity	3.00 (3.00–4.00)	4.00 (3.00–4.00)	0.47 (0.28–0.63)	<0.001

Note: Data are presented as median and interquartile range (IQR; 25th–75th percentile). Effect size (*r*) was calculated from the Z-value of the Wilcoxon signed-rank test. *p*-values for individual NSSS items were adjusted for multiple testing using the Benjamini–Hochberg false discovery rate (FDR) procedure. All items remained statistically significant after correction. CI, confidence interval; NSSS, New Sexual Satisfaction Scale.

**Table 5:** Comparison of NSSS total and subscale scores between participants from urban and rural areas at baseline and six months after surgery.

NSSS Outcome	Urban Area, *n* = 23 Median (IQR)	Rural Area, *n* = 17 Median (IQR)	Mann–Whitney U	*p*-Value
Personal Sexual Experiences—baseline	30.00 (28.00–31.00)	36.00 (34.00–37.00)	20.500	<0.001
Partner-Focused and Sexual Activity—baseline	30.00 (29.00–32.00)	37.00 (37.00–39.00)	7.500	<0.001
Total NSSS score—baseline	60.00 (58.00–62.00)	73.00 (71.00–75.00)	0.000	<0.001
Personal Sexual Experiences—6 months	32.00 (30.00–33.00)	44.00 (42.00–45.00)	1.000	<0.001
Partner-Focused and Sexual Activity—6 months	32.00 (31.00–34.00)	45.00 (43.00–46.00)	0.000	<0.001
Total NSSS score—6 months	64.00 (62.00–66.00)	88.00 (87.00–90.00)	0.000	<0.001

Data are presented as median (interquartile range, IQR). Differences between participants from urban and rural areas were tested using the Mann–Whitney U test. NSSS = New Sexual Satisfaction Scale; IQR = interquartile range.

## Data Availability

The data presented in this study are available on request from the corresponding author. The data are not publicly available due to privacy or ethical restrictions.
